# Mindfulness-based stress reduction to improve depression, pain and high patient global assessment in controlled rheumatoid arthritis

**DOI:** 10.1093/rap/rkac074

**Published:** 2022-09-05

**Authors:** Isabelle Gaboury, Patricia L Dobkin, Françoise Gendron, Pasquale Roberge, Marie-Claude Beaulieu, Nathalie Carrier, Pierre Dagenais, Sophie Roux, Gilles Boire

**Affiliations:** Department of Family Medicine, Faculty of Medicine and Health Sciences, Université de Sherbrooke, Sherbrooke, Québec, Canada; Whole Person Programme, Department of Medicine, Faculty of Medicine and Health Sciences, McGill University, Montréal, Québec, Canada; Centre Intégré Universitaire de Santé et de Services Sociaux de l’Estrie—Centre Hospitalier Universitaire de Sherbrooke (CIUSSSE-CHUS), Sherbrooke, Québec, Canada; Department of Family Medicine, Faculty of Medicine and Health Sciences, Université de Sherbrooke, Sherbrooke, Québec, Canada; Department of Family Medicine, Faculty of Medicine and Health Sciences, Université de Sherbrooke, Sherbrooke, Québec, Canada; Centre Intégré Universitaire de Santé et de Services Sociaux de l’Estrie—Centre Hospitalier Universitaire de Sherbrooke (CIUSSSE-CHUS), Sherbrooke, Québec, Canada; Centre Intégré Universitaire de Santé et de Services Sociaux de l’Estrie—Centre Hospitalier Universitaire de Sherbrooke (CIUSSSE-CHUS), Sherbrooke, Québec, Canada; Department of Medicine, Faculty of Medicine and Health Sciences, Université de Sherbrooke, Sherbrooke, Canada; Centre Intégré Universitaire de Santé et de Services Sociaux de l’Estrie—Centre Hospitalier Universitaire de Sherbrooke (CIUSSSE-CHUS), Sherbrooke, Québec, Canada; Department of Medicine, Faculty of Medicine and Health Sciences, Université de Sherbrooke, Sherbrooke, Canada; Centre Intégré Universitaire de Santé et de Services Sociaux de l’Estrie—Centre Hospitalier Universitaire de Sherbrooke (CIUSSSE-CHUS), Sherbrooke, Québec, Canada; Department of Medicine, Faculty of Medicine and Health Sciences, Université de Sherbrooke, Sherbrooke, Canada

**Keywords:** RA, patient general assessment, depression, mindfulness-based stress reduction, function, remission, coping, pain, mindfulness

## Abstract

**Objective:**

The aim was to improve distressing patient-reported outcomes (PROs) that persisted in RA patients with clinically controlled inflammation (controlled RA).

**Methods:**

In a pragmatic pilot study, we offered mindfulness-based stress reduction (MBSR), a group intervention, to controlled RA patients who had high (≥16) Centre for Evaluation Studies depression (CES-D) scores and/or patient general assessment of disease activity (PGA) at least 2/10 larger than evaluator general assessment (EGA) (PGA-EGA: Delta). Evaluations before, 6 and 12 months after MBSR included CES-D, PGA, modified HAQ, simple disease activity index (SDAI), anxiety (general anxiety disorder 7; GAD-7), coping strategies (coping with health injuries and problems; CHIP), sleep disturbance and pain. Facilitators and obstacles to recruitment and participation were identified. A subset of patients was interviewed for qualitative analysis of their experience.

**Results:**

Out of 306 screened patients, 65 were referred, 39 (60%) agreed and 28 (43%) completed MBSR. Anticipated burden, timing and frequency of group meetings, commuting issues, age extremes and co-morbidities were barriers to participation. Up to 12 months after MBSR, anxiety, depression, emotion-oriented coping, sleep and function significantly improved. Nonetheless, no significant impact was observed on pain, PGA, Delta or SDAI. The interviews revealed that benefits, including integration of effective coping strategies, were maintained.

**Conclusion:**

We addressed MBSR feasibility issues and selection of outcomes in controlled RA patients with distressing PROs. For patients who chose to participate in MBSR, lasting benefits were evident for anxiety, depression, sleep and function. Larger studies are required to evaluate the weaker impact of MBSR on RA-related pain and PGA.

Key messages

- We explored feasibility issues of mindfulness-based stress reduction (MBSR) to address negative patient-reported outcome measures in controlled RA patients.
- Improvements in depression, anxiety, sleep and emotion-oriented coping were found post-MBSR.
- MBSR in patients with controlled RA might improve function more than pain and Patient Global Assessment of disease activity (PGA).

## Introduction

RA is a chronic immune-mediated inflammatory disease. When inflammation is present (active RA), treat-to-target pharmacological interventions improve all five parameters measured by simple disease activity index (SDAI), in addition to function and patient mood [1, 2]. Fulfilling ACR/EULAR remission criteria averts, or at least limits, both structural damage and functional limitations [3]. Of the SDAI variables, patient general assessment of disease activity (PGA) remains the most refractory to attain the remission range [4], yielding a positive Patient minus Evaluator Global Assessment of disease activity (PGA-EGA) difference (Delta) despite apparently optimal control of joint and systemic inflammation (controlled RA). Large positive Deltas (+2 to +3/10) [5] are often associated with poor patient-reported outcomes (PROs), including sleep disturbances, fatigue, depression, anxiety and chronic pain [6, 7]. Owing to their known correlations, we hypothesized that improved depression would reduce PGA, Deltas and disability [8].

Reviews of mindfulness-based stress reduction (MBSR) in RA provide evidence for improvements in psychosocial factors, and possibly for pain [9, 10]. No study of MBSR to date has specifically targeted patients with controlled RA who experience distressing PROs. In this pragmatic pilot study, we initially assessed the feasibility of a randomized control trial of MBSR in such patients and subsequently examined whether reducing psychological distress would increase function, in addition to decreasing pain and the perceived impact of RA (PGA).

## Methods

### Design and recruitment

The Research Ethics Board of the Centre de recherche du Centre hospitalier universitaire de Sherbrooke (CRCHUS) approved the study (MP-31-2017-1558), originally designed as a pilot randomized control trial with allocation to immediate MBSR or to a 6-month waiting list (ClinicalTrials NCT03514355). However, owing to recruitment obstacles, randomization was not performed.

Before the start of MBSR groups, charts from RA patients scheduled for follow-up were assessed by research assistants using inclusion and exclusion criteria. After clinical assessment, physicians referred potential patients to research assistants, who confirmed eligibility and obtained written informed consent.

### Inclusion and exclusion criteria

Adult patients meeting 1987/2010 ACR RA criteria, with swollen joint count (SJC) ≤2/66 and CRP ≤8 mg/l, under stable antirheumatic treatment for ≥3 months, were included if they also had a Center for Epidemiologic Studies depression scale (CES-D) score ≥16 and/or Delta ≥2/10. Patients unable to consent or to participate in groups at prespecified times or with severe psychiatric disease were excluded.

### Standardized MBSR intervention

The group MBSR intervention was delivered in 2.5-h sessions for 8 weeks consecutively, including a 6-h retreat between classes 6 and 7 [11]. The retreat involved guided meditations, mindful movement and mindful eating, all to encourage continuity in practice. Except for the first session, each opened with a particular meditation practice, such as the body scan, sitting with awareness or mindful movement (i.e. yoga and walking meditation). Sessions included didactic exercises (e.g. identifying thoughts, emotions and body sensations associated with illness, stress management). Relevant home practices (using a workbook and recorded guided meditations) were assigned and discussed in subsequent sessions. The curriculum themes and content reflected mind–body connection principles (Supplementary Table S1, available at *Rheumatology Advances in Practice* online). To support participation, logistics were considered (i.e. time, location, in addition to transportation and parking reimbursements for each MBSR session, in addition to baseline, 6 and 12-month clinical visits).

The trained MBSR facilitator is a family physician (F.G.) under P.L.D.'s supervision to ensure treatment integrity. CDs/MP3s for practice and home practice manuals were provided to participants.

### Outcomes

Four group sessions were held between the autumn of 2017 and spring of 2019. Evaluations were conducted at baseline, 6 and 12 months from the start of the sessions; Group 4 had baseline and 6-month evaluation only. Sociodemographic and clinical data were collected, including visual analogue scales (VAS) to measure sleep disturbances, PGA and pain. At each visit, rheumatologists contributed EGA, SJC and tender joint counts, and CRP was measured. Validated and reliable questionnaires included CES-D [12], generalized anxiety disorder 7 (GAD-7) [13], modified HAQ (M-HAQ) [14], coping with health injuries and problems (CHIP) [15] and the five facet mindfulness questionnaire (FFMQ) [16].

Individual semi-structured interviews were carried out between 1 and 4 months after the end of the intervention using maximum variation sampling from the groups (until data saturation). Up to three or four participants per group were randomly selected to be interviewed.

### Analyses

Descriptive statistics were used to characterize the study population. Only data from patients who completed at least two MBSR classes were analysed. Linear mixed regression models with maximum-likelihood estimation techniques were used to evaluate whether continuous outcomes changed during follow-up. A variance components correlation matrix was used. To control for repeated measures, a random effect was added to participants. Bootstrap resampling (*Resampling* = 1000) was computed when the model residuals were not normally distributed. The generalized estimating equation was used for dichotomic outcomes with log-binomial models. Analyses were performed using IBM SPSS Statistics v.25 and SAS software v.9.4 (SAS Institute, Cary, NC, USA).

Verbatim transcripts of all audio recordings were used to conduct descriptive content analysis, based on the interactive cyclical process of data reduction, data display, conclusion drawing and verification [17]. Data were coded by two independent analysts and disagreements resolved through discussion. The NVivo software (QSR International, 2021) was used to analyse the full interview transcriptions.

## Results

Between autumn of 2017 and spring of 2019, a total of 306 charts were reviewed; 190 patients were ineligible or not offered the study, and 51 declined referral (Supplementary Fig. S1, available at *Rheumatology Advances in Practice* online). The MBSR was offered to 65 patients, 39 (60%) of whom consented, and 38 completed baseline data. Number, length or timing of groups, age extremes and commuting distance were common barriers to participation. Nine patients failed to attend, and one showed up only for the first class, leaving 28 (43%) patients for analyses. All 10 patients offered a qualitative interview agreed.

Recruited patients were predominately Caucasian women with long-standing disease (Table 1). Use of DMARDs was universal, whereas CSs (0%) and NSAIDs (11%) were rarely used. A quarter were using antidepressants. Pharmacological treatments remained stable during the study.

**Table 1.** Baseline characteristics of the 28 patients

Characteristic	Value
Age, mean (s.d.), years	61.8 (13.6)
Women, *n* (%)	24 (85.7)
Disease duration, median (IQR), years	9.8 (4.7–12.5)
Body mass index, mean (s.d.), kg/m^2^	27 (5.6)
Education, mean (s.d.), number of years	12.9 (3.2)
Caucasian, *n* (%)	27 (96.4)
Marital status, *n* (%)	
Married/living with a partner	18 (64.3)
Divorced/separated	4 (14.3)
Single	3 (10.7)
Widowed	3 (10.7)
Seropositive (*n* = 26), *n* (%)	15 (53.6)
Treatments, *n* (%)	
Biologic DMARDs	10 (35.7)
Conventional DMARDs	23 (82.1)
Prednisone	0 (0)
NSAIDs	3 (10.7)
Antidepressants	7 (25.0)

IQR: interquartile range (25th–75th percentiles).

### Mental health outcomes

**Figure 1.**
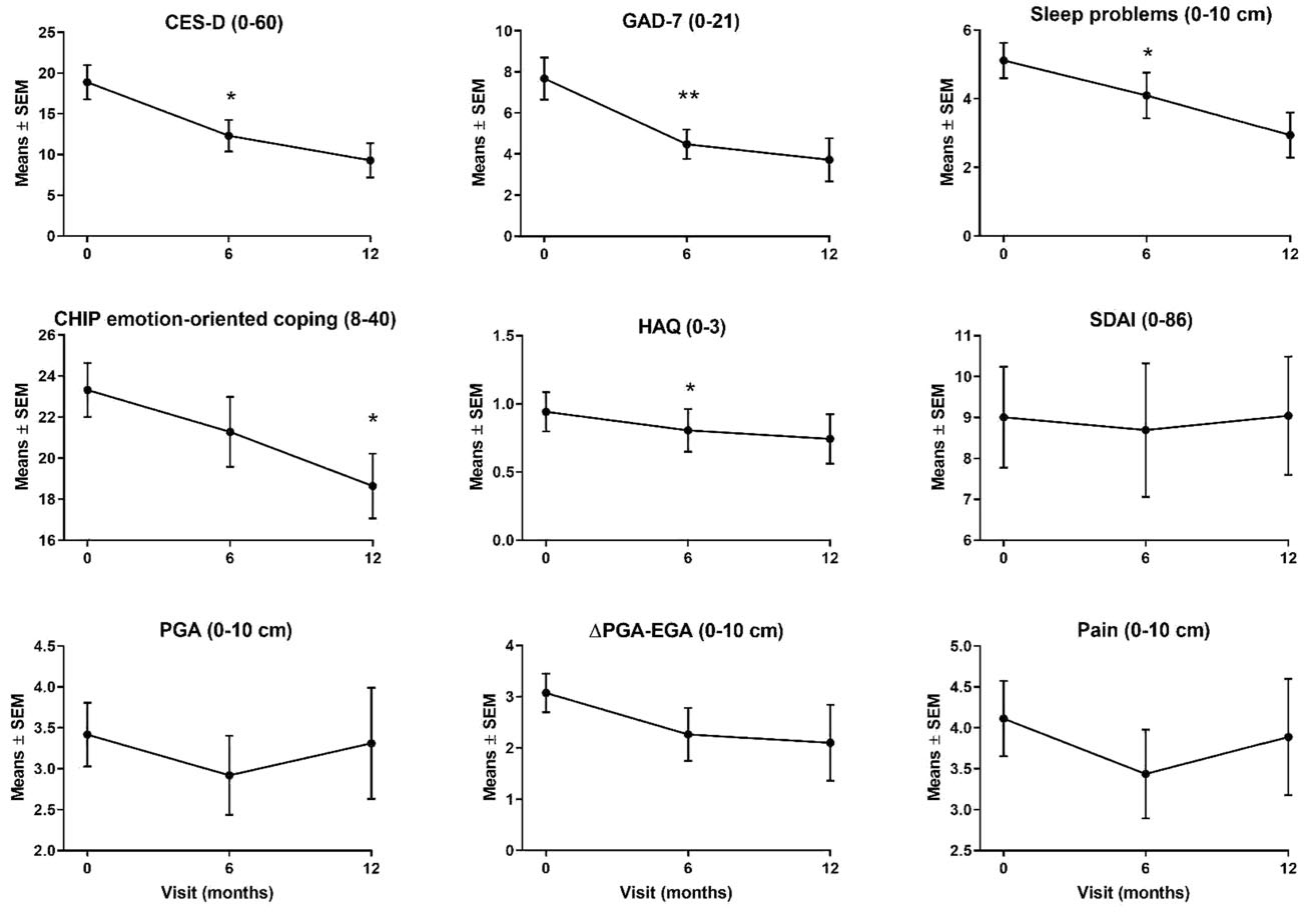
Evolution of mental health and RA-associated outcomes over time. The scales of each graph are different. CES-D: Center for Epidemiologic Studies depression scale; CHIP: coping with health injuries and problems scale; Delta: difference between PGA and evaluator general assessment of disease activity (EGA); GAD7: general anxiety disorder 7; M-HAQ: modified health assessment questionnaire; PGA: patient general assessment of disease activity; SDAI: simple disease activity index. *P < 0.05 and **P < 0.01 for 6 *vs* 0 months and 12 *vs* 6 months comparisons

Symptoms of depression, sleep disturbances and anxiety improved significantly between baseline and 6 months; these were maintained or improved at 12 months (Fig. 1; Supplementary Table S2, available at *Rheumatology Advances in Practice* online). Effect sizes and confidence intervals are shown in Supplementary Table S3, available at *Rheumatology Advances in Practice* online. The proportion of patients with CES-D ≥ 16 decreased from 67.9% at baseline to 36% (*P* = 0.01) and 11.8% (*P* = 0.002) at 6 and 12 months, respectively. Although changes in coping strategies at 6 months failed to reach significance, emotion-oriented coping improved significantly between 6 and 12 months. Post hoc exploratory analyses suggest that higher depression and anxiety scores (both found more frequently in anti-depressant users) might lead to a larger effect size of MBSR on these outcomes (data not shown).

Most interviewed patients reported that MBSR impacted their psychological well-being, and this often preceded its physical benefits. For example, patient 2 stated:

The physical benefits, you don't get them right away. It's more abstract. But you have to give yourself a chance, you have to be patient and practice. There's stuff sometimes you can't really explain to yourself, but it works, it feels good in there [pointing her head].

All interviewed patients confirmed they were still practising at least some MBSR components taught during the sessions, especially those that fitted their lifestyle and were perceived most effective at reducing stress or improving their capacity to focus or sleep while experiencing pain. More than half integrated at least one practice into their daily/weekly routine.

In addition, the opportunity to share experiences of illness with peers was often viewed as a key motivator to attend sessions, with a positive effect on mental health. For example, patient 16 stated:

We feel less alone in this situation. We feel that yes, we can improve our condition. Sometimes, we give each other tips, too. Understanding others is good for us.

### Arthritis-related outcomes

Non-significant and transient decreases in PGA, pain and SDAI were observed at 6 months. EGA increased and delta decreased at 6 and 12 months, albeit not significantly. In contrast, mean M-HAQ scores decreased from 0.9 (0.8) at baseline to 0.8 (0.8) and 0.7 (0.8) at 6 and 12 months, respectively. The decrease was significant at 6 months. Remarkably, M-HAQ ≥ 1, indicative of moderate to severe disability, decreased from 57.1% at baseline to 32.1% at 6 months (*P* = 0.007) and 26.1% at 12 months (*P* = 0.008) (Supplementary Table S2, available at *Rheumatology Advances in Practice* online).

These results are consistent with patients’ reports. Even when prompted to discuss the effects of the intervention on disease, only a minority mentioned pain reduction. However, some commented on their new relationship to pain. As patient 2 stated:

I have less pain, or maybe sometimes it's still there, but I perceive it differently. We experience it differently in our brain, so sometimes we perceive it differently. As the teacher was trying to explain to us, we should not give it all the space.

### Facets of mindfulness

The total mindfulness score (FFMQ) changed significantly at 6 months; some improvement was maintained at 12 months (Supplementary Table S2, available at *Rheumatology Advances in Practice* online). Specifically, the FFMQ components observation, description, and act with awareness increased significantly at 6 months, with a trend for non-judgmental of internal experience.

## Discussion

In this pragmatic pilot study in patients reporting depressive symptoms and/or high PGA despite controlled RA, we identified hurdles that prevented participation in MBSR group sessions. Several potentially eligible patients declined a psychosocial intervention offered by rheumatologists, seen as a joint specialist [18]. Addressing depression was less well received than reducing stress and anxiety. Using high Delta (PGA-EGA ≥2) as an inclusion criterion was simpler for rheumatologists. Interestingly, this approach identified patients who had high depression or anxiety scores for the most part. An 8-week in-person group intervention was viewed as too demanding for many. Online MBSR might appeal to these patients because it saves commute time and travel issues [19]. Nonetheless, participating patients appreciated the tools they acquired, and most integrated some mindfulness practices into their lives.

The MBSR intervention had a marked impact (lasting ≤12 months) on depression and anxiety, as expected [9, 10], and on sleep quality and emotion-oriented coping. Moreover, we observed a strong positive impact on function, the strongest predictor of health-care costs in RA [20]. However, improvements in these psychosocial PROs translated into minor or no changes in other RA-related symptoms, such as pain, PGA, Delta or DAS. Apparent failure to improve pain and PGA is puzzling, because both are highly correlated with depression, anxiety and function. Long disease duration, co-morbidities and central sensitization might explain why pain and PGA remained mostly stable, disconnected from psychosocial PROs. Although suggestive, our results need to be confirmed in larger, randomized studies.

Our study has specific strengths. Most importantly and contrary to previous trials [10, 11], RA participants had controlled disease, and therefore the effect of MBSR could be distinguished from interventions on inflammation or spontaneous variation of inflammation. Obstacles and facilitators for recruitment in future randomized control trials were identified. Follow-up was completed up to 12 months. Qualitative results complemented quantitative observations.

Nonetheless, our study has limitations. We deviated from the randomized design owing to recruitment difficulties; thus, the improvements observed cannot be ascribed to MBSR. Indeed, we lack information on the stability of PROs over time in controlled RA patients with negative PROs. Up to 40% of eligible patients declined, and close to 30% of those consenting failed to attend sessions. We propose that sharing evidence for MBSR benefits in this population might stimulate further patient interest and facilitate future recruitment. Group numbers (5 to 10 in size) were smaller than is generally recommended. Nonetheless, those who attended were afforded an opportunity to share illness experiences with peers in a safe setting. Low statistical power might explain the failure to find significant changes for some RA-related outcomes.

### Conclusion

High levels of depressive and anxiety symptoms, and perception of disease burden affect patients’ quality of life with controlled RA. This low-cost, non-pharmacological MBSR intervention was highly appreciated by participants, improved distressing PROs and provided significant long-term functional benefits. Our results suggest that MBSR might have minimal impact on pain, PGA levels and [PGA-EGA] Delta. We maintain that addressing psychological distress is of high importance in controlled RA [8], but the lack of correlation of psychosocial improvements with PGA implies that focusing on depression per se is unlikely to increase remission rates in controlled RA.

## Supplementary data

Supplementary data are available at *Rheumatology Advances in Practice* online.

## Supplementary Material

rkac074_Supplementary_Data

## Data Availability

All data are incorporated into the article and its online supplementary material.

## References

[1] Smolen JS , Landewé RBM, Bijlsma JWJ et al EULAR recommendations for the management of rheumatoid arthritis with synthetic and biological disease-modifying antirheumatic drugs: 2019 update. Ann Rheum Dis 2020;79:685–99. 31969328 10.1136/annrheumdis-2019-216655

[2] Leblanc-Trudeau C , Dobkin PL, Carrier N et al Depressive symptoms predict future simple disease activity index scores and simple disease activity index remission in a prospective cohort of patients with early inflammatory polyarthritis. Rheumatology (Oxford) 2015;54:2205–14. 26209789 10.1093/rheumatology/kev272

[3] Felson DT , Lacaille D, LaValley MP, Aletaha D. Reexamining Remission Definitions in Rheumatoid Arthritis: Considering the Twenty‐Eight–Joint Disease Activity Score, C‐Reactive Protein Level, and Patient Global Assessment. Arthritis Care & Res 2022;74:1–5. 10.1002/acr.24772 PMC11577894 34783179

[4] Vermeer M , Kuper HH, van der Bijl AE et al The provisional ACR/EULAR definition of remission in RA: a comment on the patient global assessment criterion. Rheumatology (Oxford) 2012;51:1076–80. 22302059 10.1093/rheumatology/ker425

[5] Desthieux C , Hermet A, Granger B, Fautrel B, Gossec L. Patient-physician discordance in global assessment in rheumatoid arthritis: a systematic literature review with meta-analysis. Arthritis Care Res (Hoboken) 2016;68:1767–73. 27059693 10.1002/acr.22902

[6] Kojima M , Kojima T, Suzuki S et al Depression, inflammation, and pain in patients with rheumatoid arthritis. Arthritis Rheum 2009;61:1018–24. 19644894 10.1002/art.24647

[7] Altawil R , Saevarsdottir S, Wedrén S et al Remaining pain in early rheumatoid arthritis patients treated with methotrexate. Arthritis Care Res (Hoboken) 2016;68:1061–8. 26784398 10.1002/acr.22790 PMC5129578

[8] Dobkin PL , Boire G. Controlled joint inflammation but still no remission? It’s time to attend to depressive symptoms. J Rheumatol 2018;45:585–7. 29717092 10.3899/jrheum.170801

[9] Zhou B , Wang G, Hong Y et al Mindfulness interventions for rheumatoid arthritis: a systematic review and meta-analysis. Complement Ther Clin Pract 2020;39:101088. 31957665 10.1016/j.ctcp.2020.101088

[10] Oliveira LN , Araújo ATdV, Branco JNR et al Mindfulness for patients with rheumatoid arthritis: systematic review. Research, Society and Development 2021;10:e2610212047.

[11] Santorelli SF. Mindfulness-based stress reduction (MBSR): standards of practice. 2014. https://mindfulness.au.dk/fileadmin/mindfulness.au.dk/Artikler/Santorelli_mbsr_standards_of_practice_2014.pdf (23 May 2022, date last accessed).

[12] Eaton WW , Smith C, Ybarra M et al Center for Epidemiologic Studies Depression Scale: Review and Revision (CESD and CESD-R). In Maruish ME, ed. The use of psychological testing for treatment planning and outcomes assessment: Instruments for adults. Mahwah, NJ: Lawrence Erlbaum Associates Publishers, 2004: 363–77.

[13] Spitzer RL , Kroenke K, Williams JBW, Löwe B. A brief measure for assessing generalized anxiety disorder: the GAD-7. Arch Intern Med 2006;166:1092–7. 16717171 10.1001/archinte.166.10.1092

[14] Pincus T , Summey JA, Soraci SA, Wallston KA, Hummon NP Jr. Assessment of patient satisfaction in activities of daily living using a modified Stanford Health Assessment Questionnaire. Arthritis Rheum 1983;26:1346–53. 6639693 10.1002/art.1780261107

[15] Endler NS , Parker JDA, Summerfeldt LJ. Coping with health problems: developing a reliable and valid multidimensional measure. Psychological Assessment 1998;10:195–205.

[16] Baer RA , Smith GT, Lykins E et al Construct validity of the five facet mindfulness questionnaire in meditating and nonmeditating samples. Assessment 2008;15:329–42. 18310597 10.1177/1073191107313003

[17] Sandelowski M. Whatever happened to qualitative description? Res Nurs Health 2000;23:334–40. 10940958 10.1002/1098-240x(200008)23:4<334::aid-nur9>3.0.co;2-g

[18] Heiman E , Kravitz RL, Wise BL. Rheumatologists’ approaches to diagnosis and treatment of depression. J Clin Rheumatol 2016;22:307–11. 27556237 10.1097/RHU.0000000000000383

[19] Dobkin PL. Reflections of a mindful teacher’s shift from in-person to online courses. Mindfulness 2021;12:2559–61. 34413907 10.1007/s12671-021-01722-5 PMC8364441

[20] Ohinmaa AE , Thanh NX, Barnabe C et al Canadian estimates of health care utilization costs for rheumatoid arthritis patients with and without therapy with biologic agents. Arthritis Care Res (Hoboken) 2014;66:1319–27. 24470178 10.1002/acr.22293

