# Supplementary material for: Mindfulness-based stress reduction to improve depression, pain and high patient global assessment in controlled rheumatoid arthritis
Source: Rheumatol Adv Pract. 2022 Sep 5;6(3):rkac074. doi: 10.1093/rap/rkac074 (PMC9492233; doi:10.1093/rap/rkac074)
Supplement: rkac074_Supplementary_Data [file rkac074_supplementary_data.zip › 22-039 Supplementary Table S2.docx]

**Supplementary Table S2. Evolution of outcomes over time**

|  | Baseline | 6 months | 12 months | baseline vs 6 months | | 6 vs 12 months | |
| --- | --- | --- | --- | --- | --- | --- | --- |
|  | mean ± SD | mean ± SD | mean ± SD | Estimate (95% CI) or  RR (95% CI) | p-value | Estimate (95% CI) or  RR (95% CI) | p-value |
| **Mental health outcomes** |  |  |  |  |  |  |  |
| CES-D | 18.9 ± 11.2 | 12.3 ± 9.8 | 9.3 ± 8.7 | -6.95 (-11.36 to -1.90) | **0.023** | -2.26 (-6.98 to 2.47) | 0.390 |
| CES-D ≥ 16 | 19 (67.9) | 9 (36.0) | 2 (11.8) | 0.51 (0.30 to 0.86) | **0.012** | 0.42 (0.18 to 0.99) | **0.047** |
| GAD-7 | 7.7 ± 5.3 | 4.5 ± 3.6 | 3.7 ± 4.5 | -3.18 (-5.15 to -1.22) | **0.002** | -0.59 (-2.81 to 1.64) | 0.596 |
| Sleep disturbances (0-10 cm) | 5.1 ± 2.7 | 4.1 ± 3.3 | 2.9 ± 2.9 | -1.10 (-2.06 to -0.14) | **0.026** | -0.96 (-2.04 to 0.12) | 0.081 |
| **Arthritis-related outcomes** |  |  |  |  |  |  |  |
| Morning stiffness | 53.2 ± 107.8 | 33.0 ± 54.2 | 41.1 ± 94.8 | -22.75 (-64.87 to 11.68) | 0.362 | 9.28 (-25.12 to 51.14) | 0.729 |
| HAQ | 0.9 ± 0.8 | 0.8 ± 0.8 | 0.7 ± 0.8 | -0.18 (-0.32 to -0.04) | **0.013** | -0.10 (-0.26 to 0.07) | 0.239 |
| HAQ ≥ 1, n (%) | 16 (57.1) | 9 (32.1) | 6 (26.1) | 0.58 (0.37 to 0.90) | **0.007** | 0.87 (0.67 to 1.13) | 0.259 |
| SJC68 | 0.2 ± 0.7 | 0.1 ± 0.4 | 0.5 ± 0.9 | -0.09 (-0.4 to 0.25) | 0.651 | 0.40 (-0.01 to 0.85) | 0.136 |
| TJC66 | 2.0 ± 4.3 | 1.7 ± 4.6 | 1.1 ± 1.4 | -0.32 (-2.29 to 1.09) | 0.760 | -0.37 (-1.74 to 0.92) | 0.650 |
| Pain (0-10 cm) | 4.1 ± 2.4 | 3.4 ± 2.7 | 3.9 ± 3.1 | -0.70 (-1.70 to 0.31) | 0.171 | 0.20 (-0.93 to 1.34) | 0.720 |
| PGA (0-10 cm) | 3.4 ± 2.1 | 2.9 ± 2.4 | 3.3 ± 3.0 | -0.35 (-1.36 to 0.66) | 0.490 | 0.30 (-0.80 to 1.39) | 0.589 |
| EGA (0-10 cm) | 0.3 ± 0.6 | 0.7 ± 1.0 | 1.2 ± 2.0 | 0.31 (-0.23 to 0.80) | 0.294 | 0.58 (-0.32 to 1.44) | 0.260 |
| ∆PGA-EGA (0-10 cm) | 3.1 ± 2.0 | 2.3 ± 2.6 | 2.1 ± 3.2 | -0.85 (-1.83 to 0.12) | 0.086 | -0.30 (-1.39 to 0.80) | 0.589 |
| CRP | 2.9 ± 2.7 | 3.4 ± 4.5 | 3.9 ± 4.3 | 0.47 (-1.31 to 2.18) | 0.655 | 0.57 (-2.02 to 3.39) | 0.724 |
| SDAI | 9.0 ± 6.4 | 8.7 ± 8.0 | 9.0 ± 5.8 | -0.59 (-3.54 to 2.21) | 0.720 | 0.64 (-3.04 to 4.09) | 0.732 |
| **Patient-reported outcomes** |  |  |  |  |  |  |  |
| SF-36 |  |  |  |  |  |  |  |
| Physical functioning | 58.6 ± 22.5 | 60.0 ± 23.0 | 62.3 ± 25.4 | 1.63 (-5.10 to 8.37) | 0.627 | 1.63 (-5.10 to 8.37) | 0.627 |
| Role limitations due to physical health | 38.4 ± 42.8 | 47.0 ± 41.7 | 54.2 ± 40.4 | 8.80 (-11.07 to 28.7) | 0.437 | 8.73 (-16.39 to 34.46) | 0.517 |
| Role limitations due to emotional problems | 53.6 ± 41.9 | 58.7 ± 36.4 | 70.4 ± 36.0 | 5.54 (-13.66 to 24.51) | 0.597 | 10.34 (-12.76 to 30.01) | 0.394 |
| Energy/fatigue | 44.3 ± 19.8 | 50.8 ± 18.4 | 53.1 ± 18.6 | 6.48 (-0.80 to 13.77) | 0.080 | 1.80 (-6.56 to 10.17) | 0.666 |
| Emotional well-being | 59.9 ± 17.9 | 64.3 ± 18.8 | 71.3 ± 16.7 | 4.47 (-3.98 to 12.75) | 0.360 | 7.04 (-2.53 to 17.04) | 0.225 |
| Social functioning | 60.3 ± 24.5 | 66.0 ± 20.9 | 74.3 ± 18.4 | 5.79 (-3.10 to 14.68) | 0.196 | 7.23 (-2.95 to 17.42) | 0.160 |
| Pain | 54.4 ± 23.4 | 56.3 ± 24.5 | 57.1 ± 23.6 | 2.12 (-9.01 to 13.71) | 0.791 | 3.29 (-6.96 to 15.65) | 0.637 |
| General health | 51.4 ± 20.1 | 53.4 ± 21.3 | 57.2 ± 20.0 | 3.23 (-2.91 to 9.37) | 0.295 | 3.23 (-2.91 to 9.37) | 0.295 |
| CHIP |  |  |  |  |  |  |  |
| Distraction | 26.0 ± 6.4 | 26.9 ± 6.5 | 26.7 ± 6.2 | 1.16 (-1.62 to 3.95) | 0.456 | -1.10 (-4.75 to 1.77) | 0.570 |
| Palliative | 25.0 ± 5.5 | 25.3 ± 5.5 | 24.7 ± 8.3 | 0.31 (-1.60 to 2.19) | 0.770 | -0.43 (-3.54 to 3.07) | 0.816 |
| Instrumental | 29.8 ± 5.8 | 28.4 ± 5.6 | 27.9 ± 6.3 | -1.36 (-3.45 to 0.62) | 0.235 | -0.17 (-3.20 to 3.14) | 0.930 |
| Emotional | 23.3 ± 7.0 | 21.3 ± 8.6 | 18.6 ± 6.5 | -1.68 (-3.96 to 0.61) | 0.146 | -3.50 (-6.19 to -0.80) | **0.012** |
| FFMQ |  |  |  |  |  |  |  |
| Total score | 124.4 ± 13.3 | 134.2 ± 15.2 | 131.9 ± 17.2 | 10.01 (4.64 to 15.39) | **0.001** | -2.02 (-8.26 to 4.22) | 0.518 |
| Observation | 26.1 ± 5.4 | 28.0 ± 5.7 | 27.0 ± 5.9 | 2.14 (0.44 to 3.84) | **0.015** | -0.97 (-2.93 to 0.99) | 0.325 |
| Description | 24.5 ± 4.9 | 27.0 ± 4.8 | 25.4 ± 5.7 | 2.17 (0.51 to 3.82) | **0.011** | -1.32 (-3.23 to 0.59) | 0.170 |
| Aware actions | 25.6 ± 6.8 | 28.0 ± 6.0 | 28.8 ± 5.3 | 2.44 (0.16 to 4.72) | **0.037** | 0.85 (-1.83 to 3.54) | 0.525 |
| Non-judgmental of experience | 27.5 ± 5.5 | 29.5 ± 6.8 | 28.8 ± 5.8 | 1.89 (-0.05 to 3.83) | 0.056 | -0.70 (-2.94 to 1.54) | 0.532 |
| Non-reactivity | 21 ± 2.8 | 21.8 ± 4.7 | 22.1 ± 5.2 | 1.10 (-0.93 to 3.30) | 0.394 | 0.15 (-2.19 to 2.39) | 0.923 |

CES-D: Center for Epidemiologic Studies Depression scale; BDI: Beck Depression Inventory, GAD7: General Anxiety Disorder-7, HAQ: Health Assessment Questionnaire, SJC68: 68-swolen joint count, TJC66: 66-tender joint count, PGA: Patient global assessment, EGA: Evaluator global assessment, CRP: C-Reactive Protein, SDAI: Simplified Disease Activity Index. The scales of each graph are different, SF-36: 36-Item Short Form Survey, CHIP: Coping with Health Injuries and Problems scale, FFMQ: the Five Facet Mindfulness Questionnaire. Variables are presented as mean ± standard deviation. p-values were calculated with linear mixed regression models for continuous variables.
