# Supplementary material for: Mindfulness-based stress reduction to improve depression, pain and high patient global assessment in controlled rheumatoid arthritis
Source: Rheumatol Adv Pract. 2022 Sep 5;6(3):rkac074. doi: 10.1093/rap/rkac074 (PMC9492233; doi:10.1093/rap/rkac074)
Supplement: rkac074_Supplementary_Data [file rkac074_supplementary_data.zip › 22-039 Supplementary Table S3.docx]

**Supplementary Table S3. Effect size of outcomes for differences between inclusion and 6 months and 6-12 months**

|  | Effect size (95% CI) | |
| --- | --- | --- |
|  | Inclusion and 6 months | 6 and 12 months |
| **Clinical outcomes** |  |  |
| Morning stiffness (minutes) | 0.24 (-0.16 to 0.64) | -0.22 (-0.69 to 0.25) |
| M-HAQ (0-3) | 0.49 (0.07 to 0.90) | 0.29 (-0.20 to 0.77) |
| SJC68 | 0.05 (-0.35 to 0.45) | -0.47 (-0.97 to 0.04) |
| TJC66 | 0.10 (-0.30 to 0.50) | 0.07 (-0.40 to 0.55) |
| PGA (0-10 cm) | 0.30 (-0.11 to 0.69) | -0.03 (-0.49 to 0.43) |
| EGA (0-10 cm) | -0.24 (-0.63 to 0.16) | -0.25 (-0.72 to 0.22) |
| ∆PGA-EGA (0-10 cm) | 0.37 (-0.04 to 0.77) | 0.23 (-0.24 to 0.70) |
| CRP (mg/L) | -0.08 (-0.48 to 0.32) | -0.13 (-0.64 to 0.38) |
| SDAI | 0.14 (-0.27 to 0.54) | -0.14 (-0.65 to 0.37) |
| **Patient reported outcomes** |  |  |
| CES-D | 0.62 (0.18 to 1.04) | 0.24 (-0.26 to 0.74) |
| BDI | 0.66 (0.22 to 1.09) | 0 (-0.47 to 0.48) |
| GAD-7 | 0.54 (0.11 to 0.96) | 0.19 (-0.29 to 0.67) |
| Sleep problems (0-10 cm) | 0.45 (0.03 to 0.85) | 0.40 (-0.08 to 0.88) |
| Pain (0-10 cm) | 0.29 (-0.11 to 0.69) | 0 (-0.46 to 0.47) |
| SF-36 |  |  |
| Physical functioning | -0.12 (-0.52 to 0.27) | -0.10 (-0.57 to 0.38) |
| Role limitations due to physical health | -0.21 (-0.61 to 0.19) | -0.28 (-0.76 to 0.21) |
| Role limitations due to emotional problems | -0.12 (-0.51 to 0.28) | -0.17 (-0.65 to 0.31) |
| Energy/fatigue | -0.35 (-0.75 to 0.06) | -0.23 (-0.71 to 0.26) |
| Emotional well-being | -0.21 (-0.61 to 0.19) | -0.34 (-0.83 to 0.15) |
| Social functioning | -0.23 (-0.62 to 0.17) | -0.27 (-0.75 to 0.22) |
| Pain | -0.09 (-0.48 to 0.30) | -0.24 (-0.72 to 0.24) |
| General health | -0.21 (-0.60 to 0.19) | -0.15 (-0.63 to 0.33) |
| CHIP |  |  |
| Distraction | -0.20 (-0.60 to 0.19) | 0.18 (-0.32 to 0.67) |
| Palliative | -0.12 (-0.51 to 0.28) | -0.06 (-0.55 to 0.43) |
| Instrumental | 0.35 (-0.06 to 0.75) | -0.05 (-0.54 to 0.44) |
| Emotional | 0.26 (-0.14 to 0.66) | 0.52 (-0.01 to 1.03) |
| FFMQ |  |  |
| Total score | -0.74 (-1.18 to -0.29) | 0.42 (-0.08 to 0.91) |
| Observation | -0.51 (-0.92 to -0.08) | 0.49 (-0.02 to 0.99) |
| Description | -0.60 (-1.03 to -0.17) | 0.39 (-0.11 to 0.87) |
| Aware actions | -0.37 (-0.77 to 0.04) | 0.08 (-0.39 to 0.56) |
| Non-judgmental of experience | -0.34 (-0.75 to 0.06) | 0.13 (-0.35 to 0.60) |
| Non-reactivity | -0.39 (-0.79 to 0.03) | 0.25 (-0.24 to 0.73) |

M-HAQ: Modified Health Assessment Questionnaire; SJC66: Swollen joint count on 66 joints; TJC: Tender joint count on 68 joints; PGA: Patient Global Evaluation of disease activity; EGA: Evaluator Global Assessment of disease activity; CRP: C-Reactive Protein level; SDAI: Simple Disease Activity Index; CES-D: Center for Epidemiologic Studies Depression scale; BDI: Beck Depression Inventory; GAD7: General Anxiety Disorder-7; SF-36: Short Form Health Survey; CHIP: Coping with Health Injuries and Problems; FFMQ: the Five Facet Mindfulness Questionnaire.
