# Supplementary material for: Mindfulness-based stress reduction to improve depression, pain and high patient global assessment in controlled rheumatoid arthritis
Source: Rheumatol Adv Pract. 2022 Sep 5;6(3):rkac074. doi: 10.1093/rap/rkac074 (PMC9492233; doi:10.1093/rap/rkac074)
Supplement: rkac074_Supplementary_Data [file rkac074_supplementary_data.zip › Mindfulness in RA Supplementary Figure 1.docx]

**12 months**

1 no baseline visit data

163 not eligible

51 declined referral to assistants

27 other reasons

13 refusal

13 not eligible/not available

**Used for**

**analysis**

**6 months**

**Qualitative**

**Interviews**

**Supplementary Figure 1. Flowchart.**
