# Supplementary material for: Mindfulness-based stress reduction to improve depression, pain and high patient global assessment in controlled rheumatoid arthritis
Source: Rheumatol Adv Pract. 2022 Sep 5;6(3):rkac074. doi: 10.1093/rap/rkac074 (PMC9492233; doi:10.1093/rap/rkac074)
Supplement: rkac074_Supplementary_Data [file rkac074_supplementary_data.zip › 22-038 Supplementary Table S1.docx]

**Supplementary Table S1. MBSR components and the student experiences** (from Table 1 in Garneau K, Hutchinson T, Zhao Q, Dobkin PL. Cultivating person-centered medicine in future physicians. European Journal for Person Centered Healthcare. 2013;1(2):468-477) <https://web.archive.org/web/20160819144710id_/http://bjll.org:80/index.php/ejpch/article/viewFile/688/pdf_23>

| **Class 1: Being present to yourself** | |
| --- | --- |
| **Introduction to meditation** | Meditation is not as easy as it seems to be. It allows me to be in a state of quiescent rest. Fleeting thoughts are coming and going in my mind and I become observant and “mindful” of their existence. I feel somewhat liberated from the turmoil and stresses of daily life. |
| **Self-care exercise** | I am encouraged to engage in more self-care practices, to take breaks during the day and to simply do things for enjoyment. |
| **Class 2: Perception** | |
| **Body scan meditation** | This meditation in a supine position permits better awareness of various sensations in my body. I observe and feel the rising and the falling of my breath and explore various sensations as I focus on different parts of my body. |
| **Perceiving and stress** | Each individual responds to stressors differently and this is related in part to perceptions. Stress can present itself insidiously and we explore and discuss ways to recognize it (sweaty palms, trouble thinking clearly, isolation) and deal with it effectively. |
| **Physician wellbeing** | I realize that I tend to suppress my emotions and withdraw when I face disappointment and sorrow. I learned that openness and acceptance of my feelings is the first step towards finding ways to cope better. |
| **Informal mindfulness** | I realize that I can practice “mindfulness” anytime of the day: while eating a snack, at the traffic lights, while brushing my teeth. I now pay attention to otherwise trivial moments of my life that I used to fail to notice. |
| **Class 3: Being well with what is** | |
| **Pleasant events** | This exercise cultivates the idea of “awareness” by focusing attention on pleasant moments of my life. I explore different aspects of positive experiences (physical responses, thoughts and feelings). |
| **Yoga** | The restorative yoga practiced in this course brings awareness to the different parts of my body that are tense and allows for a greater relaxation. |
| **STOP** | This mnemonic (Stop; Take a breath; Observe; Proceed) reminds me to return to the present moment when I feel overwhelmed and tense. It breaks being on “automatic pilot” and helps me focus on what it happening and needs to be done now. |
| **Triangle of awareness** | This triangle helps me conceptualize what mindfulness is: an awareness of thoughts, emotions and sensations in the context of the present moment. |
| **Class 4: Stress** | |
| **Stress and illness** | We learn how stress is directly and indirectly related to illness in ourselves and our patients. |
| **Unpleasant events** | This exercise brings me in contact with myself at a deeper level. I am inclined to ignore and overlook unpleasant events in my life. Through a conscious effort to recognize, understand and accept these disagreeable moments, I have the opportunity to make significant changes in my life. |
| **Sitting meditation** | The stillness of meditation is overwhelming at times. It is a good practice to train the mind to sustain attention. Through attempts at focusing my attention on my respiration, I acknowledge and accept this wandering mind of mine. |
| **Class 5: Mindful Communication** | |
| **Satir’s communication stances** | Virginia Satir’s communication stances clarify my feelings, e.g., perceptions of low self-confidence in the context of the healthcare system. I realize that I was often in the “placating stance”, basically renouncing myself, in an attempt to please my supervisors and the patients. |
| **Role plays** | The group explores unresolved scenarios previously experienced during clerkship and we revisit them using mindful communication. We watch those unconstructive communication habits and discover ways to change them. |
| **Walking meditation** | Walking in mindfulness brings me a sense of inner peace. I am aware of the shifting of the body from one side to the other, along with the flowing of the breath. There is no rush, no intention, nowhere to go. |
| **Class 6: Retreat** | |
| **Practice in silence (all forms of meditation learned)** | We are plunged into a day of silence, cumulating the learning from the previous classes. It initially feels like a daunting experience to spend one day without speaking. But I realize that silence can bring so much: deeper connection to our thoughts and feelings. I share privileged and memorable moments with my fellow students. |
| **Class 7: Healing/being whole** | |
| **Debrief retreat** | We discuss the personal rewards silence may bring into our lives. The silent retreat day is transformative for many of us, but it is particularly arduous and unpleasant for others. The possibility of increased rather than decreased suffering is addressed. |
| **Loving kindness meditation** | This meditation helps broaden my outlook on life, allowing me to be more open-hearted, compassionate and accepting. |
| **Mindfulness and healing** | We discuss a more holistic approach to medicine using mindfulness-based techniques. Being fully present to patients, acknowledging and accepting their illness enable us to be more compassionate. It positively affects the therapeutic relationship. Healing in medicine complements curing and we examine the importance of both. |
| **Class 8: Ending and integrating** | |
| **Making the practice your own** | The practice of mindfulness is a personal commitment, not an obligation. The tools may be used lifelong. Our instructors hope that we will remember to use those tools regularly or during times of difficulty. We talk about how to maintain and apply what we learned in the course during residency and beyond. |
| **Adherence** | Mindfulness can be carried out in multiple ways; we were taught various informal and formal practices and reviewed these. It can be applied in whatever form suits me and it can easily be integrated into my everyday life. We develop different means of daily practice and this is shared among us. |
